# Functional and Phenotypic Characterisations of Common Syngeneic Tumour Cell Lines as Estrogen Receptor-Positive Breast Cancer Models

**DOI:** 10.3390/ijms24065666

**Published:** 2023-03-16

**Authors:** Maria Lambouras, Charlotte Roelofs, Melrine Pereira, Emily Gruber, Jessica L. Vieusseux, Patrick Lanteri, Cameron N. Johnstone, Fenella Muntz, Sandra O’Toole, Lisa M. Ooms, Christina A. Mitchell, Robin L. Anderson, Kara L. Britt

**Affiliations:** 1 Breast Cancer Risk and Prevention Laboratory, Peter MacCallum Cancer Centre, 305 Grattan St, Melbourne, VIC 3000, Australia; 2 Department of Anatomy and Developmental Biology, Monash University, Clayton, VIC 3800, Australia; 3 Olivia Newton-John Cancer Research Institute, Heidelberg, VIC 3084, Australia; 4 The Peter MacCallum Cancer Centre, Melbourne, VIC 3000, Australia; 5 Sir Peter MacCallum Department of Oncology, The University of Melbourne, Melbourne, VIC 3000, Australia; 6 School of Cancer Medicine, La Trobe University, Heidelberg, VIC 3084, Australia; 7 Sydney Medical School, University of Sydney, Camperdown, NSW 2050, Australia; 8 Garvan Institute of Medical Research, Darlinghurst, NSW 2010, Australia; 9 Australian Clinical Labs, Sydney, NSW 2153, Australia; 10 Cancer Program, Department of Biochemistry and Molecular Biology, Monash Biomedicine Discovery Institute, Monash University, Clayton, VIC 3800, Australia

**Keywords:** estrogen receptor-positive, syngeneic, breast cancer cell line, mouse models

## Abstract

Estrogen receptor-positive breast cancers (ER^+^ BCas) are the most common form of BCa and are increasing in incidence, largely due to changes in reproductive practices in recent decades. Tamoxifen is prescribed as a component of standard-of-care endocrine therapy for the treatment and prevention of ER^+^ BCa. However, it is poorly tolerated, leading to low uptake of the drug in the preventative setting. Alternative therapies and preventatives for ER^+^ BCa are needed but development is hampered due to a paucity of syngeneic ER^+^ preclinical mouse models that allow pre-clinical experimentation in immunocompetent mice. Two ER-positive models, J110 and SSM3, have been reported in addition to other tumour models occasionally shown to express ER (for example 4T1.2, 67NR, EO771, D2.0R and D2A1). Here, we have assessed ER expression and protein levels in seven mouse mammary tumour cell lines and their corresponding tumours, in addition to their cellular composition, tamoxifen sensitivity and molecular phenotype. By immunohistochemical assessment, SSM3 and, to a lesser extent, 67NR cells are ER^+^. Using flow cytometry and transcript expression we show that SSM3 cells are luminal in nature, whilst D2.0R and J110 cells are stromal/basal. The remainder are also stromal/basal in nature; displaying a stromal or basal Epcam/CD49f FACS phenotype and stromal and basal gene expression signatures are overrepresented in their transcript profile. Consistent with a luminal identity for SSM3 cells, they also show sensitivity to tamoxifen in vitro and in vivo. In conclusion, the data indicate that the SSM3 syngeneic cell line is the only definitively ER^+^ mouse mammary tumour cell line widely available for pre-clinical research.

## 1. Introduction

Breast cancer (BCa) incidence has been increasing steadily for decades and now affects 1 in 7 women in the western world, making it the most prevalent cancer in women. The estrogen receptor-positive (ER^+^) BCa subtype is driving this increase in incidence. Endocrine therapies such as tamoxifen effectively treat ER^+^ BCa and are also used in the preventative setting [1]. Unfortunately, some ER^+^ BCa tumours in advanced ER^+^ BCa patients acquire resistance to tamoxifen [2], and due to the real or perceived side effects of this treatment, uptake and adherence rates of tamoxifen regimens in the preventative setting are poor [3,4,5,6]. This underscores the importance of developing additional endocrine therapies for ER^+^ BCa.

Pre-clinical studies often rely on patient-derived xenograft models, donor tissues or cells implanted in immunocompromised mice, to test next-generation endocrine therapies. There are also several genetically engineered mouse strains that develop ER^+^ mammary tumours and can be used for pre-clinical testing, including AIB1-overexpressing mice, STAT1 knockout mice and MMTV Pik3ca^H1047R^ transgenic mice [7,8,9]. However, as the tumours that form in these transgenic models exhibit a long latency, it renders pre-clinical in vivo experimentation time-consuming and costly. Alternatively, transplantable syngeneic breast cancers in mice are of great value as they allow assessment of tumours in normal stromal and immune microenvironments and can generate tumours and metastatic disease with shorter latency [10]. Two mouse mammary tumour cell lines are considered ER^+^; SSM3 and J110 [8,11]. However, there are conflicting reports of whether either of these cell lines or others (67NR, E0771, D2.0R, D2A1, 4T1.2) are genuinely ER^+^ in vitro and reliably produce ER^+^ tumours in vivo. A model of ER+ BCa should be dependent on ER signalling for growth.

Here we have tested available syngeneic cell lines for ER activity using immunohistochemical staining for Erα protein, flow cytometry, gene expression profiles and in vitro tamoxifen sensitivity. The data demonstrate that the SSM3 line is the only luminal ER^+^ and tamoxifen-sensitive cell line. SSM3 represents a reliable model for assessing the efficacy of new therapies designed at targeting ER^+^ BCa development. Our study also highlights the need for additional syngeneic models of ER^+^ BCa to represent the different molecular subcategories of ER^+^ BCa that exist in humans.

## 2. Results

### 2.1. ERα Staining in Syngeneic Tumours

**Figure 1:**
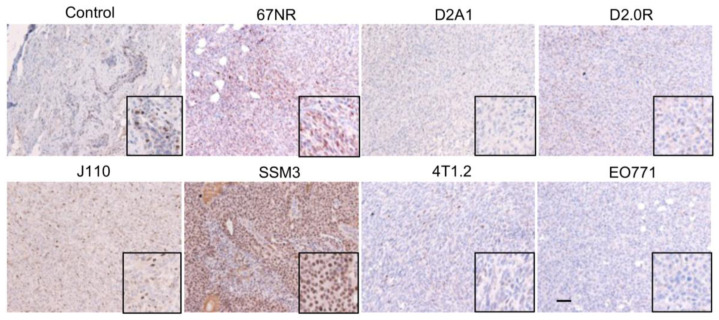
Erα expression in a collection of mouse mammary tumours. Immunohistochemical staining of Erα in epithelial tumour cells of various syngeneic mouse models. The only two cell lines producing tumours with nuclear Erα expression are the 67NR and SSM3 cells. Control: normal mouse mammary gland. Scale bar = 50 µm.

Formalin-fixed paraffin-embedded tissue sections from SSM3, 4T1.2, 67NR, J110, EO771, D2.0R and D2A1 tumours grown in mice were stained for Erα using a clinically validated antibody [12,13]. The SSM3 tumours showed strong and extensive nuclear staining (>90%) in epithelial tumour cells (Figure 1).

**Figure 2:**
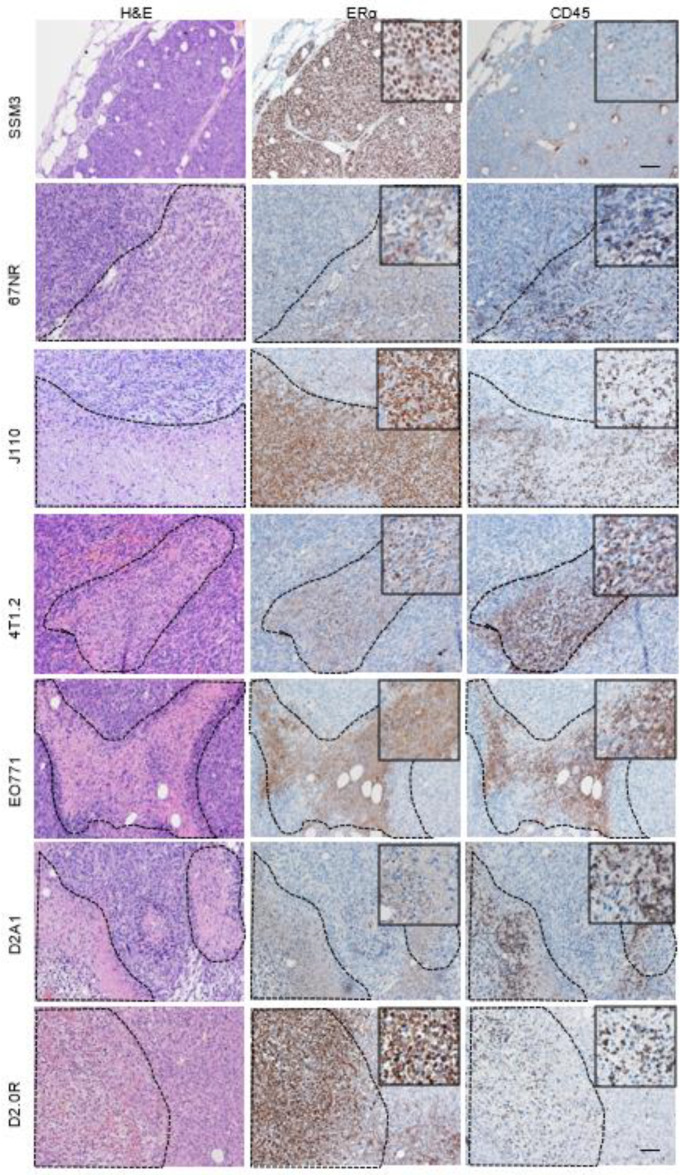
Distribution of Erα positive and CD45-positive cells in syngeneic tumours. Immunohistochemical staining of Erα and CD45 in tumours derived from different syngeneic mouse mammary tumours. ERα staining in the cytoplasm or, in the case of the J110 cell line, in the nucleus, shows concurrent staining with CD45, thus representing immune cell infiltrates. Dotted lines delineate areas of immune infiltration separate from epithelial cell counterparts. Scale bar SSM3 = 100 µm. Scale bar for all other tumours = 50 µm.

These tumours were also assessed pathologically and were classified as adenocarcinomas which are also an accurate representation of common ER^+^ tumours (Supplementary Table S1). Similarly, 67NR tumours had extensive nuclear staining but weaker staining. Scattered nuclear staining was observed in D2A1, 4T1.2 and J110 tumours. However, this staining overlapped with areas of stromal and immune infiltration, as demonstrated by CD45 immunohistochemical staining on consecutive sections (Figure 2). D2.0R and EO771 were negative for nuclear Erα expression but displayed some regions of cytoplasmic staining (Figure 1). In almost all the different tumours, cytoplasmic Erα staining was observed in regions of the tumours that had immune infiltration identified through CD45 staining, indicating, as above, cytoplasmic staining of immune cells rather than epithelial tumour cells (Figure 2).

### 2.2. SSM3 Tumours Are the Only Syngeneic Model That Responds to Tamoxifen In Vitro and In Vivo

**Figure 3:**
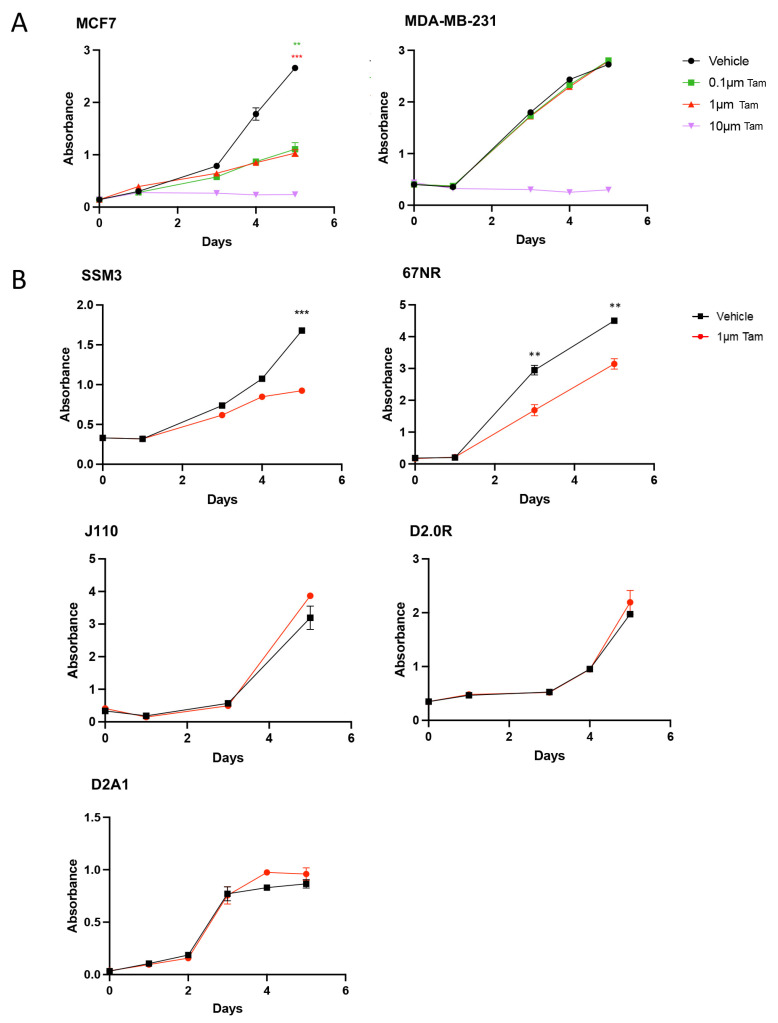
Response of syngeneic cell lines to tamoxifen treatment in vitro. Cells were incubated with either vehicle or 0.1 µM–10 µM 4-hydroxytamoxifen and proliferation was measured over 5–6 days (mean ± SEM of three to six replicate samples). (**A**) Response of commonly used human xenograft cell lines. MCF7 represents ER^+^ cells and MDA-MB-231 represents ER-cells. Both respond as expected. (**B**) Response of mouse mammary tumour lines. Tam, tamoxifen. Data shown as mean ± SEM. *** *p* ≤ 0.001, ** *p* ≤ 0.01.

Using the well-characterised human ER^+^ MCF7 cells, we conducted a dose–response to tamoxifen and showed that whilst high concentrations (10 µM) were cytotoxic, lower doses of tamoxifen could inhibit the growth of MCF7 cells (Figure 3). As a negative control, we included a dose–response in ER^-^ MDA-MB-231 cells and showed that, apart from the cytotoxic dose (10 µM), these cells were insensitive to tamoxifen treatment. All mouse tumour cell lines were tested at 1 µM, which resulted in reduced proliferation of SSM3 cells. Previously we have shown that 67NR tumours are mildly sensitive to tamoxifen and that 4T1.2 tumours are insensitive [14]. Again, we found that 67NR cells are mildly sensitive. J110 cells, D20R and D2A1 cells were all insensitive to growth inhibitory effects of tamoxifen (Figure 3). The EO771 cell line response to tamoxifen was not assessed, as it was already revealed it had no sensitivity [14].

**Figure 4:**
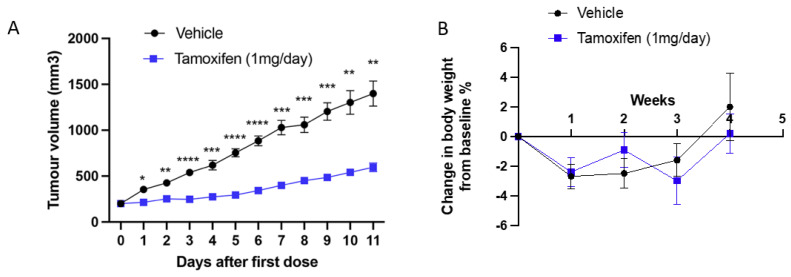
Response of SSM3 cell line to tamoxifen in vivo. (**A**) SSM3 tumour growth over time in tamoxifen-treated vs vehicle control-treated 129SvEv mice. 129SvEv mice inoculated with 100,000 SSM3 ER^+^ tumour cells and treated daily with vehicle control (*n* = 10) or 1 mg tamoxifen citrate (*n* = 7) once tumours reached 200 mm^3^. (**B**) Change in body weight from baseline of mice injected with SSM3 cells and treated with tamoxifen compared to vehicle. Data are presented as mean ± SEM. Data were analysed with a mixed-effects two-way ANOVA and Sidak’s multiple comparisons test. **** *p* ≤ 0.0001, *** *p* ≤ 0.001, ** *p* ≤ 0.01, * *p* ≤ 0.05.

To validate our results in vivo we treated mice injected with SSM3 cells in their fourth mammary fat pads with 1 mg of tamoxifen daily. Tamoxifen significantly slowed tumour growth compared to vehicle controls (Figure 4). We did not observe any toxicity issues with this dosing regimen.

### 2.3. SSM3 Cells Are the Only Syngeneic Cell Line to Express Cell Surface Markers of Luminal Mammary Epithelial Cells

**Figure 5:**
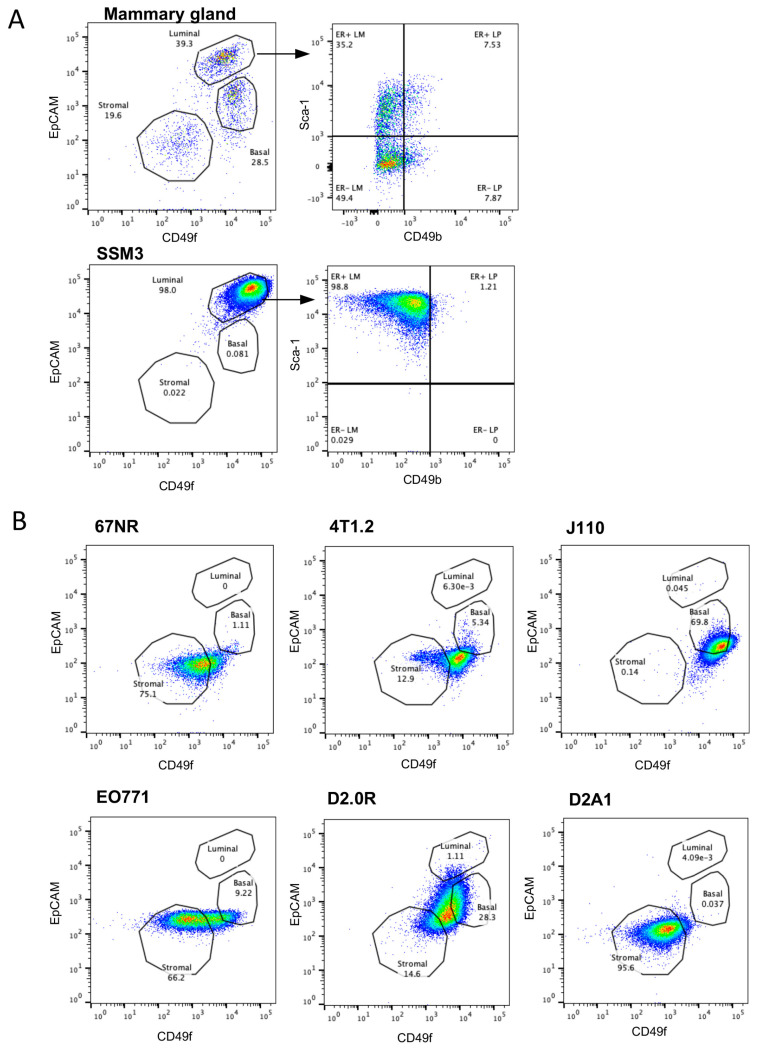
FACS profiles of syngeneic cell lines. Cells were grown in a culture and then assessed using flow cytometry markers for mammary epithelial cell subpopulations. FACS plots shown have already been gated to remove doublets, dead cells (PI positive) and lineage cells (CD45-, CD31-, TER119-). (**A**) Plots show a normal mouse mammary epithelial cell profile. EpCAM and CD49f delineate luminal, basal and stromal cells. Specific luminal cell populations can be further resolved using CD49b and Sca1. SSM3 cells are shown here as a luminal ER^+^ cell line. (**B**) Resolution of the epithelial cell subpopulations in six other mammary tumour lines. (LM, luminal mature; LP, luminal progenitor).

It is appreciated now that breast cancer arises from a dysregulation of the normal breast epithelium. The molecular definition of the cell lineages in the normal breast has been instrumental in allowing these relationships to be identified. For example, Receptor activator for nuclear factor κB (RANK)+ luminal progenitors are known to drive basal BCa development in breast cancer type 1 susceptibility protein (BRCA1) mutation carriers [15,16,17]. As ER^+^ BCa are a luminal tumour type that display luminal differentiation, we assessed which of the existing syngeneic lines were luminal in nature. Using normal mouse mammary epithelial cells as a standard plot, cell lines were assessed for the proportion of luminal, basal and stromal cells using flow cytometry (Supplementary Figure S1). Lineage gates representing endothelial (CD31), leukocyte (CD45) and erythroid (TER119) populations were used, but, as anticipated, these cell lines were all lineage-negative. Lineage-negative cells were then assessed for expression of the epithelial marker EpCAM and the luminal/basal discriminator CD49f (α6 integrin) [18,19]. The addition of CD49b (α2β1 integrin) is used as a progenitor marker and Sca-1 (Ly6a) as a marker of ER^+^ cells [20]. SSM3 cells were luminal in nature (CD49f+/EpCAM+) and found to have high expression of Sca-1, yet low expression of CD49b, which classifies them as a mature luminal cell type (Figure 5). 67NR and 4T1 cells showed a hybrid phenotype between stromal and basal, whilst J110 cells appeared basal. EO771 cells expressed stromal markers. D20R cells were again a hybrid between stromal and basal, but the populations were slightly shifted compared to the other cell lines with this phenotype. D2A1 cells were also stromal (Figure 5). Thus, the only luminal breast cancer cell line in this collection is the SSM3 line.

### 2.4. Molecular Characterisation of the Syngeneic Lines to Identify the Cell Lineage

**Figure 6:**
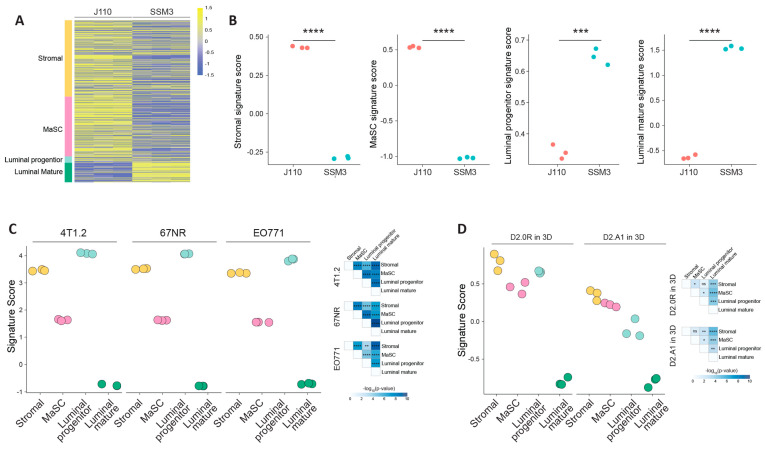
Molecular assessment of syngeneic cell lines and expression of cell-specific gene signatures. (**A**) Heatmap of genes in the MaSC, luminal progenitor and luminal mature gene signatures [16] and a stromal gene signature [15] (**B**–**D**) Gene signature scores calculated using MaSC, luminal progenitor and luminal mature genes [16] and stromal gene signature [15] in (**B**) J110 and SSM3 cells, (**C**) 4T1.2, 67NR, EO771 cells (GSE42272) and (**D**) D2A1 and D2.0R cells (GSE172882). Differences in the mean signature scores were calculated using student’s *t*-test. **** ≤ *p* 0.0001, *** ≤ *p* 0.001, ** *p* ≤ 0.01, * *p* ≤ 0.05.

Gene expression array data exist for 4T1.2, 67NR, EO771 whole tumours (GSE42272) and for D2.0R (GSE112904) and D2A1 cells (GSE12882) [14,21,22], and to complete the gene expression analysis, we performed RNAseq on J110 and SSM3 cells. Using the gene expression signatures developed by Lim and colleagues [16], we assessed whether the J110 and SSM3 cells had higher expression of certain cell epithelial cell lineages. J110 cells had high expression of stromal, mammary stem cell (MaSC) and luminal progenitor signatures, and lower expression of luminal mature genes (Figure 6A,B). SSM3 cells had high expression of the luminal mature and to a lesser extent, the luminal progenitor signature, but low expression of the stromal and MaSC signatures (Figure 6A,B). The existing gene expression array data could not be included bioinformatically in the same analysis due to the differences in the platforms used. However, when we assessed the cell lineages in 4T1.2, 67NR and EO771 tumours, they showed the highest enrichment of stromal and luminal progenitor signatures and also MaSC gene signatures, with a very low expression of luminal mature genes (Figure 6C). Gene expression data from D2.0R and D2A1 cells grown in 3D in vitro had high expression of stromal, MaSC and luminal progenitor genes and low expression of luminal mature genes (Figure 6D).

Finally, to show that the ER pathway is intact in SSM3 cells and thus mediating the effects of Tamoxifen in vitro and in vivo, we assessed whether they expressed genes, previously shown to be induced by estrogen in MCF7 cells (*PGR, CDH1, CTSD and TRIM25*). Supplementary Figure S2 shows that they are indeed increased in SSM3 compared J110 cells.

## 3. Discussion

Syngeneic mouse models are a unique preclinical tool that is now heavily used to assess the impact of emerging therapies on cancer growth rates in animals with intact immune systems. This is particularly important now that the critical role of the immune system in cancer has been revealed and immune checkpoint inhibitors have been added to the arsenal of anti-cancer therapies [23].

Previously it was reported that EO771 cells are luminal in nature due to the expression of ERβ, PR and ErbB2 in RT PCR data and that they are tamoxifen-sensitive [24]. However, we show that neither the cell lines nor the tumours produced by EO771 cells are luminal or positive for ER^+^. In agreement with Johnstone et al. 2015 [14], we have also demonstrated their highly mammary stem cell and stromal genomic signatures, but no luminal signature. In the 67NR cells that have been reported previously as a non-metastatic ER^+^ BCa cell line [25], we found weak ER staining and a partial sensitivity to tamoxifen, consistent with the conclusions reported in Johnstone et al. 2015 [14]. 4T1.2 cells are generally considered to be TNBC, but we (and others) have shown that whilst insensitive to tamoxifen, they do express ER mRNA [14], indicating that transcript expression alone cannot be used to define preclinical ER^+^ models. J110 cells were developed from Amplified In Breast 1 (AIB1)-overexpressing mice and were initially deemed ER^+^ cells, as per IHC staining [11]. However, several studies have subsequently shown that they are non-responsive to tamoxifen both in vitro and in vivo [11,26,27]. We note that our studies show that J110 cells alone do not have a luminal phenotype using flow cytometry. In addition, nuclear and cytoplasmic staining of ERα in some tumours generated from J110 cells may be confusing the field regarding ER classification. However, the positive staining correlates with areas of immune infiltration, as delineated by CD45^+^. It is known that some immune cells in the mammary gland, such as macrophages, are ER^+^, but are not routinely accounted for in the pathological classification of hormone receptor status in breast tumours [12]. Our research shows that apart from SSM3 cells, none of the other mammary tumour cell lines had all the features of truly ER^+^ cells; which are nuclear ER positivity by IHC in epithelial cells, a luminal cell surface phenotype (Sca1+), a luminal molecular subtype and sensitivity to tamoxifen exposure.

SSM3 cells were derived from signal transducer and activator of transcription 1 (*STAT1)*-deficient mice [28]. STAT1 is a transcription factor required for interferon signalling [29]. It is lost or significantly diminished in 45% of human ER^+^/PR+ breast cancers compared to normal breast tissue [8]. Mice lacking STAT1 developed ER^+^ breast tumours with a long latency (~23 months) [8]. Three tumour cell lines have been established from these mice (SSM1, SSM2 and SSM3), with SSM3 cells showing ERα positivity and responsiveness in mice to oophorectomy (removing their source of endogenous estrogen) [8]. In line with these findings, we confirm that the SSM3 cell line is a model of ER+ breast cancer.

Whilst the SSM3 model is robust, it does not recapitulate the heterogenous nature of ER^+^ BCa, as not all BCa are STAT1-deficient. The most common mutations in ER^+^ BCa are *PIK3CA*, *MLL3*, *MAP3K1*, *GATA3*, *MAPK24* (and ER itself) [30,31]. Further studies need to focus on developing additional syngeneic mouse models of ER^+^ BCa that recapitulate the clinical disease. Such models need careful definition of their phenotype, including an extensive analysis of their luminal cell properties and their responsiveness to endocrine therapy. The presence of transcripts for ERα is insufficient for defining luminal ER+ tumours.

## 4. Materials and Methods

### 4.1. Cell Lines

The EO771 cell line was derived from a spontaneous mammary tumour in a C57BL/6 mouse [32]. The 67NR and 4T1 cell lines were derived from different subpopulations of a single mammary tumour that arose in a BALB/c/C3H mouse [33]. The 4T1.2 variant was derived from 4T1 cells as described previously [34]. The SSM3 cells (kindly gifted by Robert Schreiber, Washington University) were generated from mammary tumours that arose in 129SvEv STAT1-deficient mice [8]. J110 cells (kindly gifted by Myles Brown, Dana Farber Cancer Institute) were derived from transgenic mice that expressed AIB1 cDNA under the control of the MMTV LTR [9,11]. D2.0R and D2A1 cells (kindly gifted by Dalit Barkan, University of Haifer, Haifer, Israel) were derived from clones of a BALB/C mouse with a D2 hyperplastic alveolar nodule (HAN) [35,36].

67NR and 4T1.2 mammary tumour cells were maintained in Eagle’s minimum essential medium (alpha modification) supplemented with 5% (*v/v*) foetal bovine serum (FBS) (SAFC Biosciences, Brooklyn, Victoria, Australia) and 1% (*v/v*) penicillin-streptomycin. EO771 cells were maintained in Dulbecco’s modified Eagle’s medium (DMEM) containing HEPES (20 mM) (Gibco, Billings, MT, USA) supplemented with 10% (*v/v*) FBS, penicillin (100 IU/mL) and streptomycin (100 μg/mL) (Gibco). SSM3 cells were maintained in DMEM/F12 + Hepes 15 mM, 10% FBS, 2% L-glutamine, 0.05 mM β-mercaptoethanol, 0.3 μM hydrocortisone (Sigma-Aldrich, Saint Louis, MO, USA), 5 μg/mL insulin, 10 ng/mL holo-transferrin. J110 cells were cultured in DMEM/F-12 (Corning, Corning, NY, USA) containing 5% FBS, 5 µg/mL insulin and 0.1 µg/mL hydrocortisone. MCF7 cells were maintained in RPMI supplemented with 10% (*v/v*) FBS, 1% (*v/v*) penicillin-streptomycin and 10 μg/mL insulin. MDA-MB-231 cells were maintained in L-glutamine Dulbecco’s modified Eagles’ medium (DMEM) (Gibco, Billings, MT, USA) supplemented with 10% FBS and 1% penicillin/streptomycin. All cells were cultured at 37 °C in 5% CO_2_ (*v/v*) in air and were maintained in culture for a maximum of 4–5 weeks. The only exception to this was the SSM3 cells that are maintained in 10% CO_2_.

### 4.2. Isolation, Staining and Flow Cytometric Analysis of Normal and Transformed Mouse Mammary Cells

Primary mouse mammary gland cells were used as controls for flow cytometry and were isolated using mechanical and enzymatic disaggregation as described previously [37]. The epithelial subpopulations were isolated from the disaggregated samples by flow cytometry as detailed previously [18,19,38]. Hematopoietic lineage cells were stained with anti-CD45-PE-Cy7, anti-TER119-PE-Cy7 and anti-CD31-PE-Cy7 (BD Biosciences, Franklin Lakes, NJ, USA). Cells were simultaneously stained with mammary-specific lineage markers, anti-Epcam BV421(BD Biosciences), anti-CD49f-APC (Biolegend, San Diego, CA, USA), anti-Sca-1-PE (BD Biosciences), anti-CD49b-FITC (Biolegend, San Diego, CA, USA) and propidium iodide (Sigma). Cells were analysed on a Fortessa-X20 flow cytometer (Becton Dickinson, Franklin Lakes, NJ, USA). Linear density contour plots were used to describe flow cytometry gates. Fluorescence-minus-one control gates defined marker-negative populations.

### 4.3. Immunohistochemical Analysis

Tissue sections were stained using a standard protocol. In brief, slides were heated for antigen retrieval by pressure cooker treatment in 0.01 M sodium citrate buffer, pH 6.0 (125 °C for 3 min, 90 °C for 10 s). Sections were then blocked in 3% H_2_O_2_ before blocking in 5% normal goat serum or CAS-Block Histochemical Reagent (Thermofisher, Waltham, MA, USA). Slides were incubated with primary antibodies and were incubated overnight at 4 °C. Biotin-conjugated goat anti-rabbit or anti-mouse secondary antibodies (Dako, Carpinteria, CA, USA) were used at a 1:250 or 1:300 dilution for 1 h at room temperature. Specific primary–secondary antibody complexes were detected using ABC reagent (Vector Laboratories, CA, USA) and visualised using a 3,3′-diaminobenzidine peroxidase substrate kit (Vector Laboratories, Burlingame, CA, USA). Sections were counterstained with hematoxylin, dehydrated and mounted. The primary antibodies were as follows: mouse monoclonal anti-human ERα (1:50, clone 6F11 Abcam, Cambridge, UK) and rat anti-mouse CD45 (1:300, CM5p, Novocastra, Newcastle upon Tyme, UK). Staining was viewed on a BX-53 light microscope (Olympus, Macquarie Park, NSW, Australia). Images were acquired using Spot software version 5.0 (Spot Imaging Solutions, Diagnostic Instruments Inc., Bentleigh East, VIC, Australia).

### 4.4. In Vitro Assessment of Tamoxifen Sensitivity

Proliferation assays were completed using the sulforhodamine B (SRB) colorimetric assay as described previously [39]. Cells were seeded into 96-well plates at relevant cell densities suited for each cell line. Proliferation was assessed in the presence of 0.1, 1 and 10 µM (MCF7, MDA-MB-231) or 1 µM (SSM3, 67NR, J110, D2.0R, D2A1) 4-hydroxytamoxifen (4-OHT) dissolved in ethanol and diluted to a final ethanol concentration of 1% (*v*/*v*). Statistical analysis was completed on GraphPad using a two-way ANOVA to determine significant differences.

### 4.5. In Vivo Assessment of Tamoxifen Sensitivity in the SSM3 Mouse Model

All animal work was completed with approval from the Peter MacCallum Cancer Centre Animal Ethics Committee (E594). 129SvEv mice were injected with 100,000 SSM3 BCa cells in the right fourth inguinal mammary fat pad. 17β-estradiol pellets (0.3 mg) were implanted subcutaneously in the dorsal flank of each mouse. Tumour volume was monitored using digital callipers and once tumours reached a volume of 200 mm^3^ (palpable), mice were administered either tamoxifen citrate (Sigma) at 1 mg/day or vehicle control via daily subcutaneous injection.

### 4.6. RNAseq Analysis

J110 and SSM3 cells were grown to 70–80% confluency, detached and washed in PBS. RNA was extracted using Trizol lysis following the manufacturer’s instructions (Invitrogen) and then a phenol, chloroform extraction was performed. RNA was precipitated using isopropanol, cleaned with ethanol and then solubilised in RNase-free water. The quality and quantity of RNA was assessed on the Agilent 2200 Tapestation (Agilent Technologies, Santa Clara, CA, USA). The Molecular Genomics Core at the Peter MacCallum Cancer Centre used the QuantSeq 3′ mRNA-seq Library Prep Kit for Illumina (Lexogen 015) according to the manufacturer’s instructions to generate the sequencing libraries from 500 ng of purified RNA (*n* = 3 biological replicates of each cell line). We then generated 75 bp single end reads with a depth of ~6 million reads per sample using the Illumina NextSeq500. Sequencing reads were demultiplexed using bcl2fastq (v 2.17.1.14), low quality (Q < 30) reads removed, and trimmed at the 5′ and 3′ ends using cutadapt (v 2.1) [40] to remove adapter sequences and poly-A-tail-derived reads, respectively. Sequencing reads were mapped to the mouse reference genome (mm10) using HISAT2 (v 2.0.4) [41] and counted using the featureCounts command of the Subread package (v 2.0.0) [42]. Read normalisation and differential gene expression analysis were performed in R (v 4.1.0) using R packages limma (v 3.48.3) [43] and EdgeR (v 3.34.0) [44]. The data from SSM3 and J110 cells have been deposited on Gene Expression Omnibus GSE226910. GSE112094 [21] and GSE172882 [22] were mapped using STAR (v 2.5.3a) [45], counted using Subread (v 2.0.0) [42] and voom normalised using limma (v 3.48.3) [43]. Normalised expression from GSE42272 [14] was collapsed to the median gene level. Signature scores using MaSC, luminal progenitor and luminal mature gene signatures [16] and stromal gene signature were calculated as previously defined by Lim et al. 2009 [15]. Briefly, the signature score is calculated for each sample and is the average log expression of the genes in the signature weighted by the direction and magnitude (logFC) [15,16]. R packages pheatmap (v 1.0.12), ggplot2 (v 3.3.5) and ggrepel (v 0.9.1) were used to generate figures.

## Data Availability

All the data are provided in the article and include GSE files for new RNAseq data and also the GSE reference numbers for the publicly available datasets we have used.

## Supplementary Materials

The following supporting information can be downloaded at: https://www.mdpi.com/article/10.3390/ijms24065666/s1.
