# Supplementary material for: Functional and Phenotypic Characterisations of Common Syngeneic Tumour Cell Lines as Estrogen Receptor-Positive Breast Cancer Models
Source: Int J Mol Sci. 2023 Mar 16;24(6):5666. doi: 10.3390/ijms24065666 (PMC10053941; doi:10.3390/ijms24065666)
Supplement: Supplementary file 1 [file ijms-24-05666-s001.zip › ijms-2258513-supplementary.pdf]

Supplementary Table S1: Pathological assessment of syngeneic tumours

Tumour	*Pathology*	*Background strain*
SSM3	Adenocarcinoma	129/SvEv
E0771	Anaplastic Carcinosarcoma	C57Bl/6
4T1.2	Sarcoma	Balb/C
D2.OR	Carcinosarcoma	Balb/C
67NR	Sarcoma	Balb/C
J110	Sarcoma	FVB/N
D2A1	Carcinosarcoma	Balb/C

Supplementary Figure S1: FACS gating strategy

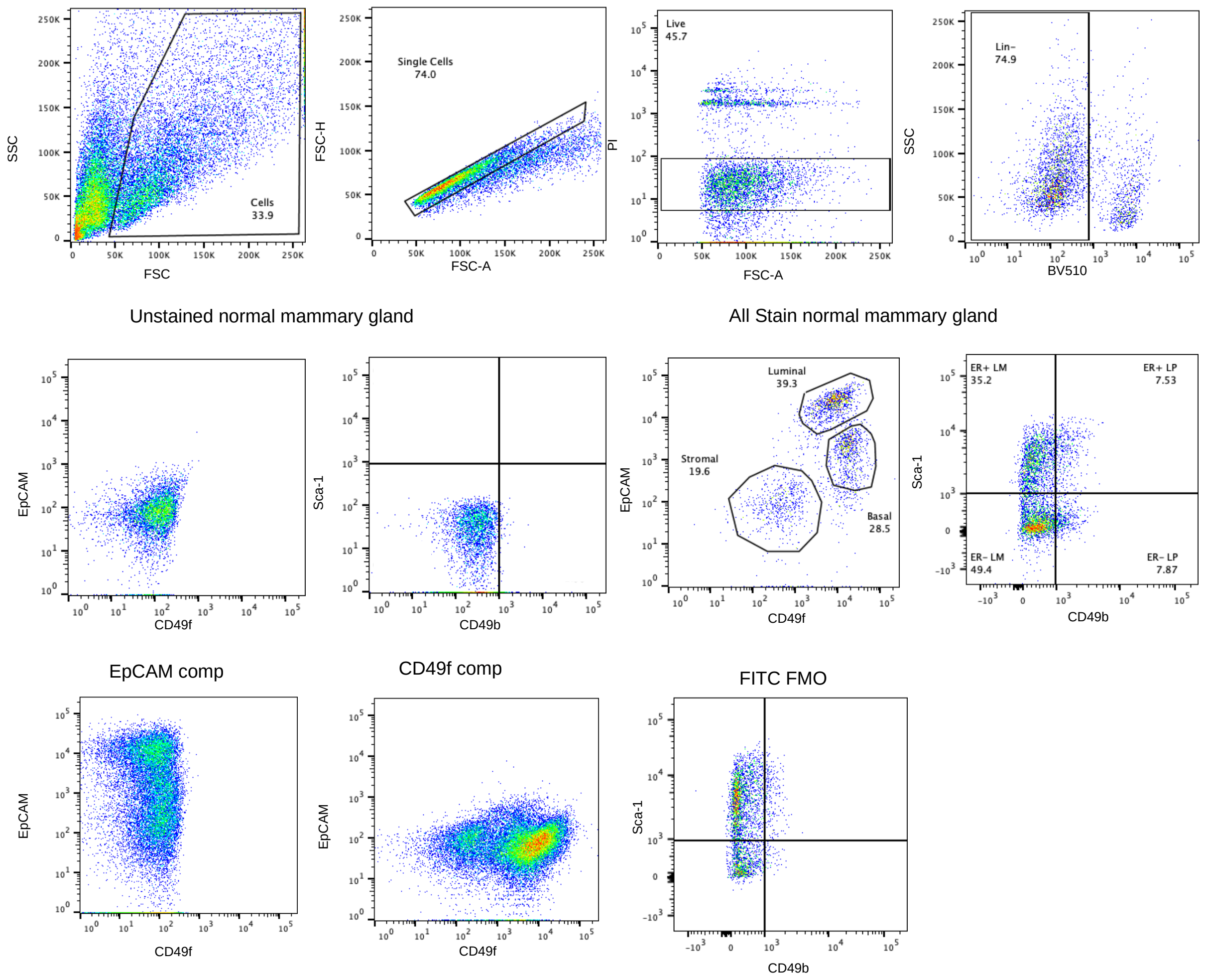

Supplementary Figure S1: Representative plots of the FACS gating strategy. After gating for our cells (FSC vs SSC), singlets (FSC-A vs FSC-H), live cells (propidium iodide) and excluding lineage cells (Ter119, CD31, CD45) we are then able to delineate our epithelial gates using a combination of unstained controls, comp controls and FMOs.

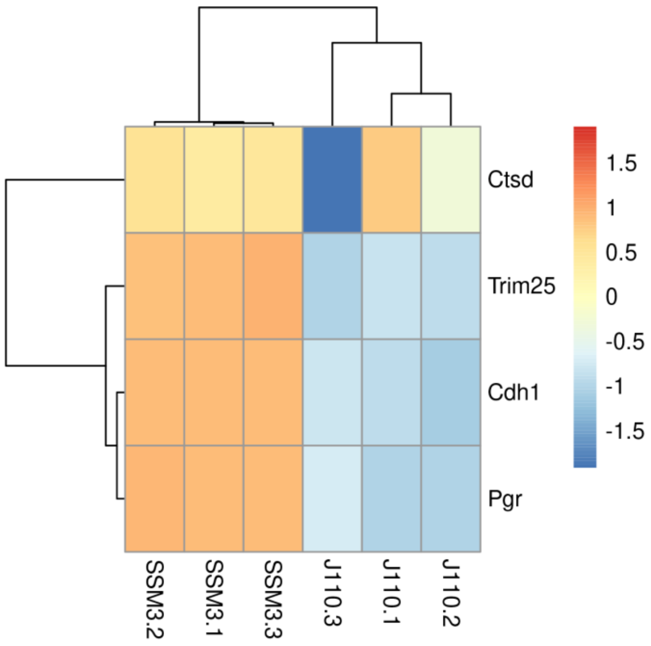

Supplementary Figure S2: Heatmap of genes induced by estrogen in MCF7 cells when assessed in SSM3 and J110 gene expression data. Scale shows increased relative expression in red and decreased expression in blue.
